# Repetitive and restricted behaviors and interests in autism spectrum disorder: relation to individual characteristics and mental health problems

**DOI:** 10.1186/s12888-023-04766-0

**Published:** 2023-05-23

**Authors:** Sara Jasim, Adrienne Perry

**Affiliations:** Department of Psychology, York University, Toronto, ON Canada

**Keywords:** Autism spectrum disorder, Repetitive behaviors, Psychopathology, Co-occurring mental health problems

## Abstract

**Background:**

Although repetitive and restricted behaviors and interests (RRBIs) may interfere with well-being and functioning in autistic individuals, research on their relation to sex, age, cognitive level, and mental health problems remains unclear. Much of the research to date has used broad categorizations rather than specific categorizations of RRBIs to examine the difference in RRBIs between individuals. The purpose of this study was to explore, in different groups of individuals, the presence of specific RRBI subtypes, and to examine the association of specific RRBI subtypes with symptoms of internalizing and externalizing behaviors.

**Methods:**

Secondary data analyses were conducted using the Simons Simplex Collection dataset, which included 2,758 participants (aged 4 to 18). Families of autistic children completed the Repetitive Behavior Scale–Revised (RBS-R) and the Child Behavior Checklist.

**Results:**

Across all RBS-R subtypes, results revealed no sex differences. Older children showed higher rates of Ritualistic/Sameness behaviors than younger children and adolescents, whereas younger and older children showed more Stereotypy than adolescents. Additionally, lower cognitive level groups showed higher rates of RBS-R subtypes except for Ritualistic/Sameness. After controlling for age and cognitive level, RBS-R subtypes accounted for a substantial amount of variance in internalizing and externalizing behaviors (23% and 25%, respectively). Specifically, Ritualistic/Sameness and Self-Injurious Behavior both predicted internalizing and externalizing behaviors, whereas Stereotypy only predicted internalizing behavior.

**Conclusions:**

These findings have key clinical implications that emphasize not only the consideration of sex, age, and cognitive level, but also specific RRBIs and co-occurring mental health problems, when assessing for ASD and designing individualized interventions.

## Introduction

Repetitive and restricted behaviors and interests (RRBIs; [1]) have long been a core deficit in Autism Spectrum Disorders (ASD). The presentation of ASD symptoms, including RRBIs, tends to vary based on individual characteristics, such as sex, age, cognitive level, and co-occurring mental health problems [2–5]. Given the significant variation in ASD symptoms, it is important to understand their differential associations with individual characteristics as well as their relationships with co-occurring mental health problems, which are highly prevalent in autistic individuals [3]. Thus, in the present study, RRBIs and their association with individual characteristics and co-occurring mental health problems were examined to further understand the variation in ASD profiles.

RRBIs are highly frequent, rigid repetitive behaviors that are often accompanied by a strong desire for sameness [6]. RRBIs, though variable, are often broadly categorized and may fail to capture the variability in different repetitive behaviors [5]. For example, RRBIs have been categorized into two classes of behaviors: lower-order RRBIs (L-RRBIs) and higher-level RRBIs (H-RRBIs; [4, 5]). L-RRBIs refer to behaviors that are not cognitively mediated and appear aimlessly repetitive and stereotypic, such as hand flapping or repetitive manipulation of an object. Higher-order RRBIs (H-RRBIs) refer to the more complex behaviors that are cognitively mediated and specialized, such as circumscribed interests or rigidity in complex non-functional routines [4, 5]. Similarly, Uljarević et al. (2021) [7] conducted a psychometric analysis of RRBIs using 4 different measures of RRBIs and revealed three distinct RRBIs: (1) repetitive-motor behaviors, (2) insistence on sameness, and (3) unusual and circumscribed interests. Uljarević and colleagues (2021) [7] emphasized the importance of using quantitative measures that reveal distinct groupings of repetitive behaviors to capture subtleties in symptom presentations.

Dichotomous or broad classifications of RRBIs oversimplify the conceptualization of RRBIs and must be utilized cautiously as important differences within a class may be obscured by its broad classification [4, 5]. For example, although both considered L-RRBIs, the impacts of stereotyped behavior and self-injurious behavior on an individual are vastly different. To address this ambiguity, Bodfish et al. (2000) [8] developed the Repetitive Behavior Scale-Revised (RBS-R), which conceptually grouped RRBIs into six distinct rationally derived subtypes: stereotypic behavior (e.g., hand flapping), compulsive behavior (e.g., lining up objects), ritualistic behavior (e.g., counting objects before brushing teeth), sameness behavior (e.g., distress upon changes in furniture), self-injurious behavior (e.g., head banging), and restricted interests (e.g., fascination with traffic lights). Subsequently, Lam and Aman (2007) [9] found that an empirically derived a 5-factor model of RRBIs was a better fit, which combined ritualistic and sameness into one subscale. Classifying RRBIs into separate, empirically-driven subtypes allows for a more comprehensive and specific assessment of these behaviors.

RRBIs are not unique to ASD or atypical development but are also exhibited early in life by typically developing (TD) infants [10]. Thelen’s (1981) [10] developmental theory provides a description of RRBIs as behaviors that serve multiple functions in development, with motor stereotypies implicated in an infant’s transition from uncoordinated movements to more controlled and voluntary movements. The development of neuromuscular control from uncoordinated movements is also essential in the development of speech production, revealing the importance of RRBI’s adaptive and cascading effects. In TD infants, RRBIs begin to decrease over time when their purpose is attained, such as skill acquisition [11]. In autistic children, however, RRBIs persist and have been shown to impede learning and contribute to adjustment difficulties, thus negatively impacting an individual’s quality of life [12]. Hence, it is not surprising that long-lasting RRBIs are associated with co-occurring mental health problems in individuals on the autism spectrum [13, 14]. In addition, the presentation of RRBIs has sometimes been found to vary as a function of an individual’s sex, age, and cognitive level. However, results have been ambiguous with inconsistent findings in the literature, partly due to the use of different RRBI categorizations, with few studies examining individual RRBIs [15].

### Sex

Frazier et al. (2014) [16] examined sex differences in autism symptoms using data from the SFARI Simons Simplex Collection (SSC; version 14.1), which consisted of 304 females and 2,114 males on the autism spectrum ranging in age from 4 to 18 years. RRBIs were assessed via the Autism Diagnostic Observation Scale (ADOS), ADI-R, and RBS-R, and they found that females demonstrated significantly fewer repetitive behaviors as assessed via the ADI-R and less restricted interests assessed via the RBS-R. In contrast, Antezana and colleagues (2019) [17], using a sample of 615 autistic individuals (507 boys) ranging in age from 3 to 18 years, assessed RRBIs via individual RBS-R items and found that females engaged in more compulsive, sameness, restricted, and self-injurious behaviors than boys, who demonstrated more stereotyped and circumscribed interests. Notably, the authors emphasize that it is unclear whether these elevated rates of RRBIs are representative of the female ASD phenotype or related to the heightened levels of co-occurring mental illness found in autistic girl group. A more recent study by Siracusano et al. (2021) [18] examined a sample of 210 children and youth on the autism spectrum (145 males) ranging in age from 3 to 18 years and found no sex differences on any of the RBS-R subtypes. Thus, there are clear discrepancies in the literature on the differences in RRBI presentations between males and females. Further research is needed using a large sample size of autistic males and females who do not have significant mental illness. Different findings could be attributed to sample differences, especially variations in the male/female split of the sample with most studies having a disproportionately higher number of males than females [19]. There were also different methodologies, sample sizes, and different alpha levels used in these studies. Finally, it has been suggested that, because ASD criteria were developed based primarily on the presentation of boys, symptom presentations displayed by girls may be qualitatively different and missed in standard diagnostic measures (McFayden et al., 2019) [20].

### Age

Most studies on the relationship between age and RRBIs are cross-sectional and have slightly varying findings [19]. Mirenda et al. (2010) [21] found different patterns in age-related RRBI patterns using a sample of young children ranging in age from 24 months to 64 months. Specifically, they found small positive correlations between age and RRBIs, such that older age was associated with more RRBIs, except for self-injurious behavior, which was endorsed less in older age groups.

Lam and Aman (2007) [9] found age-related patterns in RBS-R subtypes using a sample of 307 individuals (253 males) with a wide age range (3 to 48 years). Specifically, their descriptive data suggested that young autistic individuals ( < = 5 years old) displayed more stereotypic movements than older individuals (6 + years old) and individuals 13 years and older displayed more ritualistic and sameness behaviors. The rates of compulsive behavior, self-injurious behavior, and restricted interests were comparable across the sample. Based on Lam and Aman’s (2007) [9] findings on age-dependent patterns of various RRBI subtypes, Esbensen et al. (2009) [22] investigated age-related differences in RRBIs using a sample of participants ranging in age from 2 to 62. Overall, they found a negative association between age and RBS-R scores, such that older age was associated with fewer RRBIs. Interestingly, they also found that younger individuals (2–21 years) displayed more L-RRBIs, whereas older individuals (over 21) displayed more H-RRBIs.

Three short-term longitudinal studies have examined age-related changes in RRBIs in younger children. Harrop et al. (2014) [2] examined the developmental trajectory of RRBIs of 49 preschool children and toddlers, with a mean age of 45 months, over the span of 13 months via observational methods. This study did not yield any significant changes in RRBIs in that relatively short time span. Richler et al. (2010) [23] conducted a short-term longitudinal study on the development of RRBIs in autistic children over the span of seven years, starting at age 2. They found that the levels of L-RRBIs remained relatively stable over time and that H-RRBIs, initially low in early childhood, increased over time. These findings suggest that RRBIs are not entirely stable and may change or develop over time. More recently, Courchesne et al. (2021) [14] longitudinally examined the prevalence of RRBIs and its associations with NVIQ and age across three timepoints: preschool age of diagnosis, at age 6, and lastly at age 11. Using the ADI-R classifications of RRBIs, the researchers found that most RRBIs decreased with increasing age.

Based on the patterns of results in ASD samples with a wide age range, younger individuals tend to display more RRBIs than older individuals. When examining the types of RRBIs, younger children engage in more L-RRBIs whereas older individuals engage in more H-RRBIs. However, the findings differ depending on the age ranges included in the samples, methodologies employed, and RRBI measurements. Further, it is important to consider the cognitive level of the samples and differentiate between actual age effects versus developmental level effects (i.e., a 3-year-old may display higher L-RRBIs but an older individual with a mental age of 3 years may show similar RRBIs). Much of the research to date has looked at age-related patterns using broad categorizations of RRBIs; thus, more research is needed to elucidate the chronological age-related patterns across specific subtypes of RRBIs.

### IQ/cognitive level

The heterogeneity of RRBIs can also vary depending on cognitive level. Research studies examining broad categorizations of RRBIs showed that individuals with a low IQ and low adaptive functioning engaged in more severe and frequent L-RRBIs than individuals with higher IQ [22, 24].

Gabriels et al. (2005) [24] examined the associations between nonverbal IQ (NVIQ) and RBS-R subtypes in a small sample of 14 autistic children (mean age = 10 years). Based on the distribution of the participants’ nonverbal IQ scores, they were split into “High” NVIQ (NVIQ > = 97) and “Low” NVIQ (NVIQ < = 56); there were no participants with IQs between 57 and 98. They found a negative association between NVIQ and total RBS-R scores, such that the lower NVIQ group generally displayed more RRBIs. However, when examining the RBS-R subtypes individually, they only found a significant difference in Sameness behaviors between the two NVIQ groups. More specifically, those in the “Low” NVIQ group had a higher prevalence of Sameness than those in the “High” NVIQ group. There were no other differences in the prevalence of RBS-R subtypes between the two groups. In contrast, Bishop et al. (2006) [23] examined the RRBIs of a sample of 830 autistic children ranging in age from 15 months to 11 years. Using the ADI-R, the researchers found that NVIQ was positively related to circumscribed interests, such that higher NVIQ was associated with a higher prevalence of circumscribed interests. Interestingly, they also found that the strength of the association of NVIQ increased with age; specifically, in children older than 36 months, the negative influence of NVIQ became stronger across all RRBIs assessed except for circumscribed interests. Thus, NVIQ negatively predicted the presence of RRBIs, and this prediction became stronger in older age groups. These findings lent support to the idea of distinct RRBI classes with differential associations depending on age and cognitive level.

The longitudinal study by Richler et al. (2010) [23] examined the RRBIs of a sample of 192 children on the autism spectrum and 22 children not on the spectrum who were initially assessed at age 2, then ages 3, 5, and 9. Richler and colleagues (2010) [23] found that, in the ASD sample, L-RRBIs were high and remained high over time, whereas H-RRBIs were low initially and increased over time. However, they also found that having a higher NVIQ at age 2 was associated with lower levels of L-RRBI with improvement in its severity over time. Courchesne et al. (2021) [15], in the longitudinal study mentioned above, did not observe any significant associations in RRBI patterns as a function of NVIQ alone. However, they did find that NVIQ and age interacted significantly, such that higher NVIQ predicted the stability in Sameness behaviors over time while lower NVIQ predicted an increase in those behaviors.

In sum, the evidence discussed suggests that key developmental factors (i.e., age and IQ) influence the presentation of RRBIs in the ASD population and both must be considered when designing a study examining RRBIs and associated clinical characteristics. However, with the nature of IQ being age-corrected, its use as a measure of cognitive ability in individuals with neurodevelopmental conditions has been called into question [25, 26]. Thus, using non-age-corrected measures (e.g., raw scores or mental age) of intellectual ability is recommended [26]. Therefore, it will be preferable to clarify the role of cognitive level on RRBIs, independent of age, by using a raw score such as mental age.

### Co-occurring Mental Health Problems

Autistic individuals may experience a wide range of co-occurring emotional and behavioral problems [3, 13, 27, 28]. A systematic review on rates of mental health problems in the ASD population reported that approximately 70% of autistic individuals have one or more mental illness and 50% are diagnosed with multiple mental illnesses [27]. These mental health problems often overlap with ASD symptom presentation, making it difficult to determine whether they are symptoms of true mental illness or a reflection of RRBIs. Mental health problems in ASD have only recently been acknowledged as potentially distinct from, rather than as a symptom of, ASD [3]. Increased efforts to inhibit individuals on the spectrum from engaging in RRBIs, a core feature of ASD, may potentially contribute to the substantial rates of mental health problems in this population. In turn, co-occurring mental health problems may alter the presentation of ASD symptoms and have implications for treatment and prognosis.

Research has revealed that RRBIs negatively impact an individual’s well-being, which may have cascading negative impacts on the development of mental health problems [12–14, 24, 29]. For example, attempting to inhibit or stop an autistic individual from engaging in RRBIs is associated with agitation, anxiety, or even aggression [7, 9]. With the phenomenology of RRBIs still largely unknown, it is evident that these behaviors are associated with unfavorable outcomes for individuals and their families that go above and beyond ASD alone [12, 30]. Thus, it is important to elucidate the relationship between RRBIs and co-occurring mental health problems.

Georgiades et al. (2011) [31] investigated the shared phenotypes of psychiatric symptoms and core diagnostic domains of ASD. They found substantial overlap between RRBIs and emotionally reactive behaviors, anxious/depressed, attention problems, and aggressive behavior. These associations were independent of intellectual functioning, warranting further research into the link between RRBIs and emotional and behavioral problems. Another study by Stratis and Lecavalier (2013) [14] found associations between specific RRBI subtypes and co-occurring mental health problems, some of which were moderated by level of adaptive functioning, a developmental level variable based on informant report, that is strongly correlated with cognitive level. Specifically, they found that ritualistic/sameness behaviors were positively predictive of anxiety and Oppositional Defiant Disorder severity, regardless of adaptive functioning level. Restricted interests were negatively predictive of depressive severity and compulsive behaviors were positively predictive of Attention Deficit/Hyperactive Disorder severity [14]. There were also some interactions with adaptive level. When adaptive functioning was high, self-injurious behavior was positively predictive of anxiety and depressive severity. When adaptive functioning was low, ritualistic/sameness behaviors were positively predictive of depressive severity, and self-injurious behavior was negatively predictive of anxiety and depressive severity.

Taken together, these findings suggest that RRBIs are implicated as predictors in the later development of mental health problems and some subtypes may be dependent on (i.e., be moderated by) the developmental level of an individual. Interestingly, Garcia-Villamisar and Rojahn (2015) [13] found that co-occurring mental health problems and stress mediated the relationship between RRBIs and other autistic symptoms. They suggest that by targeting treatment of mental health problems and reducing stress, RRBI severity may decrease, leading to overall symptomatology improvement. By examining associated mental health problems, researchers can further clarify the nature of RRBIs in ASD and consider their prevalence when designing treatments [14].

Not only are RRBIs often stigmatizing and deemed as socially inappropriate, but they also often occupy an individual’s waking hours, interfere with learning opportunities, and affect family functioning [12, 24]. Jang and Matson (2015) [32] found that higher ASD severity, including higher RRBI severity, predicted more co-occurring mental health problems, which not only altered the presentation of ASD symptomatology but also led to subsequent difficulties in functioning. Further, a longitudinal study by Baribeau and colleagues (2021) [33] tracked children’s trajectory of insistence on sameness and anxiety and found that children who experienced increased severity of insistence on sameness over time also experienced an identical trajectory of increased anxiety. Thus, Baribeau et al. (2021) [33] revealed the possibility of predicting a child’s anxiety trajectory based on RRBI severity. These findings should be interpreted while keeping in mind the difficulty in distinguishing mental illness from RRBIs, as they may be identical in presentation but are separate underlying constructs (e.g., see Lahey et al., 2017) [34]. Further research is needed to elucidate the association of RRBIs and mental health problems to gain a better understanding of various symptom profiles [35]. This knowledge can help inform initial assessment, prognosis, and individualized treatment plans. Given the influence of age and IQ on RRBI patterns, studies should control for these variables while examining the link between RRBIs and mental health problems.

Overall, the objective of this study was to elucidate the different specific types of RRBIs in a large sample of children and youth on the autism spectrum, who are heterogeneous in age and cognitive level, and to explore the association of RRBIs with mental health symptoms. The two main domains of mental health are Internalizing Behavior (e.g., anxiety) and Externalizing Behavior (e.g., aggression) [36]. These two domains represent mental health symptoms but do not definitively indicate mental health diagnoses. This is particularly important as research on emotional and behavioral functioning in ASD is typically gathered via informant- or self-report measures, and not comprehensive clinical assessments. Thus, this study will examine broad domains of mental health that represent the presence of symptoms.

The first objective was to examine the presence of individual RRBIs in children and youth on the autism spectrum based on various sex, age, and cognitive level variables and to look for differences across groups within each of those categorical variables. Only one study has examined non-corrected cognitive level in relation to individual characteristics [7]. Due to the ambiguous results in the literature regarding patterns of RRBI in different sex and age groups, and the dearth of sufficient literature examining cognitive level, no directional hypotheses were provided regarding RRBI patterns in each of the groups.

The second objective was to explore the relationship of RRBIs to mental health problems, taking developmental factors of age and cognitive level into account. We anticipate based on previous literature that RRBIs will be associated with mental health problems; however, this objective was also exploratory since we set out to examine specific RRBI subtypes and operationalized mental health problems as internalizing and externalizing behavioral difficulties.

## Methods

***Participants***. Participants consisted of 2,758 children and youth (86% males; age range 4–18 years) from the Simons Simplex Collection (SSC) of the Simons Foundation for Autism Research Initiative (SFARI) who met criteria for ASD. All participants had a nonverbal mental age of 24 months or greater. Some of the exclusion criteria included complicated medical histories (e.g., prematurity), motor or sensory impairments, or genetic syndromes. Detailed descriptions of the exclusion criteria can be found through the SFARI website, https://www.sfari.org/. The dataset was missing cognitive level data from 6 participants; thus, cognitive level data from 2.752 participants were used in the appropriate analysis.

***Procedure***. We applied for and were granted permission to use the Simons Simplex Collection (SSC) of the SFARI database for secondary data analyses in the present study. The SSC dataset was a main project of SFARI, which consists of data collected from autistic individuals from simplex families. Ethics approval was obtained from the York University Human Participants Review Committee. Information on the SSC data collection process and procedures can be found through the SFARI site (https://www.sfari.org/). See Table 1 for family demographics.

**Table 1:** Demographic Information of Sample

	*n*	%
Annual household income		
< $20,000	247	9.2
$21,000-$50,000	520	19.3
$51,000-$80,000	844	31.4
$81,000-$100,000	410	15.3
$101,000-$130,000	80	3.0
$131,000-$160,000	141	5.3
> $161,000	443	16.5
Total	2685	100.0
Mother’s education		
Less than high school	6	0.2
Some high school	23	0.8
High school diploma/GED	238	8.1
Some post-secondary	613	21.8
Associate degree	226	8.0
Bachelor’s degree	1020	35.7
Graduate degree	711	25.4
Total	2837	100.0
Father’s education		
Less than high school	13	0.5
Some high school	54	1.9
High school diploma/GED	341	12.1
Some post-secondary	540	19.2
Associate degree	187	6.7
Bachelor’s degree	882	31.4
Graduate degree	793	28.2
Total	2810	100.0
Parent’s marital status		
Never married	64	2.3
Married	2563	89.7
Separated	39	1.4
Divorced	171	6.0
Total	2837	100.0

For the first objective in this study, individuals were grouped by age and cognitive level. Age was divided into early childhood (4 to 5:11 years), middle childhood (6 to 10:11 years), and adolescence (11 to 18 years), as set logically by the SFARI team based on preschool-aged children, elementary school-aged children, and middle and high school-aged youth. Cognitive level was grouped by the present authors, based on the distribution of the data and clinical judgement. Categories were constructed using the raw mental age scores (in months) to 120+, 84–119, 49–83, and < = 48. See Table 2 for descriptive statistics for these categorical variables. The distribution of cognitive level between the three age groups significantly differed, such that older age groups had higher cognitive level scores, which follows a logical pattern given older children may have higher cognitive skills given their developmental stage.

**Table 2:** Descriptive Statistics for Categorical Variables

	*n*	%
Age groups		
Early childhood	639	23.2
Middle childhood	1361	49.3
Adolescence	758	27.5
Total	2758	100
Sex		
Males	2383	86.4
Females	375	13.6
Total	2758	100
Cognitive Level groups		
120+	615	22.3
84–119	612	22.2
49–83	921	33.5
<=48	604	22
Total	2752	100

For the second objective in this study, continuous measures of age (in years) and a non-age-corrected measure (mental age in months) of cognitive level were used.

### Measures

***Repetitive Behavior Scale-Revised*** [8]. The RBS-R is a parent-report measure of the type and severity of RRBIs. If one item was missing from any of the first three RBS-R factors, the average of the remaining items for that scale was imputed; however, if two or more items were missing, the data for that scale were omitted for that individual. If even one item was missing from the latter two scales, which had fewer items, then the respective scale data was omitted from analysis for that individual. Lam and Aman’s RBS-R scoring system was used in this study due to its empirically supported psychometric properties. The five Lam and Aman (2007) [9] subscales include: (1) Stereotyped Behavior, (2) Self-Injurious Behavior, (3) Compulsive Behavior, (4) Ritualistic/Sameness Behavior, and (5) Restricted Interests. The internal consistency of the RBS-R subscales in this sample revealed Cronbach’s alpha values ranging from good to acceptable (see Table 3 for RBS-R psychometrics).

**Table 3:** Psychometric Properties of the RBS-R Scales

RBS-R Factors	Number of items	*M*	*SD*	Cronbach’s α
R/S	12	8.31	6.45	0.871
SIB	8	2.09	2.86	0.718
STEREO	9	6.31	4.79	0.795
COMP	6	2.98	3.00	0.711
REST	3	3.71	2.46	0.665

*Note.* R/S = Ritualistic/Sameness; SIB = Self-Injurious; STEREO = Stereotypy; COMP = Compulsive; REST = Restricted Interests

***Achenbach System of Empirically Based Assessment*** [37]. The Child Behavior Checklist (CBCL) is a parent-report measure often used to assess broad psychopathology. The two *T* scores used as dependent variables in the present study were Internalizing Behavior, which includes Anxious/Depressed, Withdrawn, and Somatic Complaints and Externalizing Behavior, which includes Rule-Breaking and Aggressive Behaviour. Item-level responses were not available; thus, internal consistency was not assessed.

***Cognitive level measures***. Cognitive data were based on one of several different IQ measures (Differential Ability Scales-Second Edition, Mullen Scales of Early Learning, Weschler Intelligence Scale for Children-Fourth Edition, and Wechsler Abbreviated Scale of Intelligence). For purposes of this study, IQ scores from all tests were converted into mental age scores (Mental Age = (IQ x chronological age) /100), which were then used as a measure of cognitive level. In this sample, cognitive level ranged from 9.7 months to 345.6 months (*M* = 87.8, *SD* = 48.8).

### Statistical analyses plan

IBM SPSS Statistics V27 was used for all data analyses in the present study. First, preliminary data analyses were conducted in which descriptive statistics were run for all our variables of interest, grouped by age, sex, and cognitive level. This summary data enabled us to scan the dataset for any missing and impossible values, determined via the range of scores, as well as any potential input errors from data entry. The distributions and descriptive statistics were computed for all variables and there were no significant concerns. Due to the very large sample size and to be as conservative as possible, the alpha value was set as *p* < .001 to determine significance. Further, Games-Howell post hoc tests were used when appropriate to account for the possibility of unequal population variances in various subgroups.

The first objective on the level of each RRBIs in age (early childhood, mid childhood, and adolescence), sex, and cognitive level (120+, 84–119, 49–83, and < = 48) subgroups was determined via a *t* test (for sex) and two one-way ANOVA analyses for age and cognitive level subgroups, followed by Games-Howell post hoc tests as appropriate.

The second objective on the association between RBS-R subscales and Internalizing and Externalizing Behavior *T* scores was examined via correlational and regression analyses. Thus, the distributions of all variables were examined. The CBCL *T* scores of Internalizing and Externalizing Behavior were normally distributed, whereas all RBS-R mean subscale scores appeared positively skewed. However, due to the large sample size, deviations from normality did not invalidate the results (Field, 2018) [38]. The linearity and homogeneity of variance assumptions were met, determined via inspecting the relationship of each RBS-R subscale with Externalizing and Internalizing Behavior. The lack of substantial correlations between predictors, as demonstrated via the correlational analysis, indicates a lack of collinearity in the data, satisfying the multicollinearity assumption. Further, Durbin-Watson for Externalizing and Internalizing Behaviors revealed independence of residual values. Finally, no influential cases were found (Cook’s Distance < 1).

## Results

### Objective 1: levels of RRBIs across individual characteristics

The first objective of the present study was to determine any significant differences of RRBI severity across the groups within each of the child variables (i.e., sex, age, and cognitive level, all used as categorical variables). A *t* test was used to determine any difference between males and females. Two one-way ANOVAs were conducted to determine any differences within the three age groups and the four cognitive level groups, which were followed by Games-Howell post hoc comparisons.

***RRBIs in Males and Females***. The *t* test revealed similar rates of RRBIs in males and females with no significant differences on any of the RBS-R scales (using our conservative *p* value). See Table 4 for the *T* scores between males and females for each of the RBS-R scales.

**Table 4:** Mean Scores of RBS-R Severity in Males and Females for Each Subscale

	Sex						*t*		*p*	*d*	
	Male			Female							
	*n*	*M*	*SD*	*n*	*M*	*SD*					
R/S	2370	0.69	0.54	373	0.71	0.53	0.60	0.548		0.03	
SIB	2369	0.26	0.34	374	0.30	0.37	2.05	0.041		0.11	
STER	2361	0.71	0.53	372	0.63	0.51	-2.64	0.008		− 0.15	
COMP	2374	0.50	0.50	375	0.52	0.49	0.95	0.344		0.05	
REST	2380	1.25	0.82	373	1.17	0.81	-1.74	0.083		− 0.10	

*Note.* R/S = Ritualistic/Sameness; SIB = Self-Injurious; STER = Stereotypy; COMP = Compulsive; REST = Restricted Interests

* *p* < .001

***RRBIs in Each Age Group.*** RBS-R scores were compared across the three age groups (early childhood, middle childhood, and adolescence). See Table 5 for RBS-R descriptive statistics within each age group and Table 6 for the group comparison results. Significant between-group differences were found in the Ritualistic/Sameness and Stereotypy scales of the RBS-R. In the Ritualistic/Sameness scale, post hoc tests revealed that the middle childhood group demonstrated more Ritualistic/Sameness than the early childhood group with a small effect size (*M* = 0.74, *SD* = 0.55 vs. *M =* 0.62, *SD* = 0.50). On the Stereotypy scale, individuals in the early childhood (*M =* 0.80, *SD* = 0.55) and middle childhood group (*M =* 0.72, *SD* = 0.53) demonstrated more Stereotypy than those in the adolescence group with a small effect size (*M =* 0.57, *SD* = 0.50). No between-group differences were found for the Self-Injurious, Compulsive, or Restricted Interests scales.

**Table 5:** Descriptive Statistics of RBS-R Severity for Each Subscale Across Age Groups

RBS-R Factors	Age Groups								
	Early Childhood			Middle Childhood			Adolescence		
	*n*	*M*	*SD*	*n*	*M*	*SD*	*n*	*M*	*SD*
R/S	636	0.62	0.50	1356	0.74	0.55	751	0.69	0.54
SIB	635	0.24	0.36	1354	0.27	0.35	754	0.27	0.37
STER	637	0.80	0.55	1349	0.72	0.53	747	0.57	0.50
COMP	638	0.49	0.49	1356	0.51	0.51	755	0.49	0.49
REST	639	1.19	0.85	1359	1.29	0.82	755	1.19	0.80

*Note.* R/S = Ritualistic/Sameness; SIB = Self-Injurious; STER = Stereotypy; COMP = Compulsive; REST = Restricted Interests

* *p* < .001.

**Table 6:** RBS-R Severity across Age Groups

RBS-R Factors	*F*	*df1*	*df2*	*p*	η^2^	Games-Howell Post Hoc
R/S	11.19*	2	2740	< 0.001	0.01	M > E
SIB	1.13	2	2740	0.32	0.00	-
STER	33.63*	2	2730	< 0.001	0.02	E, M > A
COMP	0.55	2	2746	0.58	0.00	-
REST	5.24	2	2750	0.01	0.00	-

*Note.* R/S = Ritualistic/Sameness; SIB = Self-Injurious; STER = Stereotypy; COMP = Compulsive; REST = Restricted Interests. E = Early Childhood; M = Middle Childhood; A = Adolescence.

* *p* < .001

***RRBIs in Each Cognitive Level Group***. Similar comparisons were made across the four cognitive level groups (120 m+, 84-119 m, 49-83 m, and < = 48 m). See Table 7 for RBS-R descriptive statistics within each cognitive level group. Significant between-group differences were found in all scales of the RBS-R.

**Table 7:** Descriptive Statistics of RBS-R Severity for Each Subscale Across Cognitive Level Groups

RBS-R Factors	Cognitive Level Groups								
	120 m+		84-119 m		49 – 83 m			<=48 m	
	*n*	*M(SD)*	*n*	*M(SD)*	*n*		*M(SD)*	*n*	*M(SD)*
R/S	611	0.69(0.54)	607	0.76*(*0.56)	919	0.71(0.53)		601	0.62(0.53)
SIB	614	0.24(0.34)	609	0.22(0.33)	917	0.25(0.36)		598	0.34(0.40)
STER	608	0.47(0.43)	607	0.63(0.51)	913	0.71(0.49)		600	1.00(0.56)
COMP	612	0.43(0.47)	611	0.47(0.51)	921	0.53(0.50)		600	0.55(0.50)
REST	614	1.11(0.76)	611	1.24(0.81)	921	1.29(0.84)		602	1.30(0.82)

More specifically, as shown in Table 8, within the Ritualistic/Sameness scale, Games-Howell post hoc tests revealed that the 84-119 m group demonstrated greater severity on this scale than the < = 48 m group. Within the Self-Injurious scale, post hoc tests revealed that the < = 48 m group demonstrated greater severity than the 120 + m group, the 84-119 m group, and the 49-83 m group. These between-group differences within the Self-Injurious subscale were significant with a small effect size. Within the Stereotypy scale, those in the < = 48 m group demonstrated greater severity than those in the 49-83 m group and those in the 84-119 m. Further, the 84-119 m group showed greater severity than the 120 m + group. Overall, the lower the cognitive level, the higher the levels of Stereotypy. Within the Compulsive subscale, the 49-83 m group and the < = 48 m group showed greater severity than the 120 m + group. Similarly, within the Restricted Interests subscale, those in the 49-83 m group and the < = 48 m group showed greater severity than the 120 m + group.

**Table 8:** RBS-R Severity across Cognitive Level Groups and Post Hoc Comparison

RBS-R Factors	*F*	*df1*	*df2*	η^2^	Post Hoc
R/S	6.36*	3	2734	0.01	2 > 4
SIB	12.40*	3	2734	0.01	4 > 1, 2, 3
STER	116.28*	3	2724	0.11	4 > 3 > 2 > 1
COMP	7.62*	3	2740	0.01	3, 4 > 1
REST	6.72*	3	2744	0.01	3, 4 > 1

*Note.* 1 = 120 m+, 2 = 84–119 m, 3 = 49–83 m, 4 = <=48 m

* *p* < .001

### Objective 2: association between RRBIs and internalizing and externalizing behaviors

The second objective was to determine the association of each of the five RBS-R scales with Internalizing and Externalizing Behavior. Two three-step hierarchical regressions were conducted to examine the associations between the five RBS-R scales and each of the two dependent variables, Internalizing Behavior and Externalizing Behavior, controlling for age (Step 1) and cognitive level (Step 2), using continuous scores for all variables.

Correlations were examined prior to the regression analysis between all continuous variables of interest, including the covariates (age and cognitive level), independent variables (RBS-R scales), and dependent variables (CBCL Internalizing and Externalizing *T* scores). See Table 9 for the correlation matrix. Overall, there were no substantial correlations between any of the independent variables that would invalidate the regression analyses.

**Table 9:** Correlation Matrix

	Age	CL	EB	IB	R/S	SIB		STER	COMP	REST
Age	-									
CL	0.70**	-								
EB	− 0.14	− 0.10	-							
IB	0.05	0.15	0.52**	-						
R/S	0.03	0.02	0.42*	0.41*	-					
SIB	0.03	− 0.09	0.35*	0.27	0.35*		-			
STER	− 0.17	− 0.33*	0.30	0.24	0.44*		0.42*	-		
COMP	− 0.01	− 0.10	0.25	0.25	0.56**		0.31*	0.47*	-	
REST	− 0.01	− 0.09	0.28	0.27	0.60**		0.29	0.46*	0.47*	-

*Note.* CL = Cognitive Level, EB = Externalizing Behavior, IB = Internalizing Behavior, R/S = Ritualistic/Sameness; SIB = Self-Injurious; STER = Stereotypy; COMP = Compulsive; REST = Restricted Interests

* medium correlation (> 0.3)

** strong correlation (> 0.5)

***Prediction of Internalizing Behavior***. A three-step hierarchical regression analysis was conducted for Internalizing Behavior (see Table 10). Age was entered in the first block, cognitive level in the second block, and the five RBS-R predictor variables in the third block. In the final model, the significant predictors were age, cognitive level, Ritualistic/Sameness, Self-Injurious Behavior, and Stereotypy. Thus, lower age with a higher cognitive level and higher scores on the Ritualistic/Sameness, Self-Injurious Behavior, and Stereotypy subscales were associated with greater levels of Internalizing Behavior. However, Compulsive Behaviors and Restricted Interests were not significant predictors.

**Table 10:** Three-Step Regression for Internalizing Behavior – Age, Cognitive Level, and RBS-R

Model	B	SE.B	β	*t*	*R* ^2^	∆*R*^2^	η_p_^2^
1 (Constant) Age	59.02 0.011	0.502 0.004	0.051	117.64 2.65	0.003	0.003	0.02
2 (Constant) Age Cognitive level	58.99 − 0.026 0.046	0.495 0.006 0.005	− 0.114 0.233	119.20 -4.25* 8.71*	0.030	0.027	0.01 0.00
3 (Constant) Age Cognitive level R/S SIB STER COMP REST	52.32 − 0.038 0.063 5.106 3.701 2.112 0.058 0.410	0.550 0.005 0.005 0.419 0.513 0.408 0.413 0.258	− 0.170 0.322 0.288 0.139 0.117 0.003 0.035	95.11 -7.03* 12.62* 12.19* 7.22* 5.18* 0.14 1.59	0.234	0.204	0.02 0.06 0.05 0.02 0.01 0.00 0.00
* F* (7, 2689) = 117.54*							

*Note.* R/S = Ritualistic/Sameness; SIB = Self-Injurious; STER = Stereotypy; COMP = Compulsive; REST = Restricted Interests

* *p* < .001

***Prediction of Externalizing Behavior***. A similar three-step hierarchical regression analysis was conducted to examine each RBS-R scale’s predictive power on Externalizing Behavior while controlling for the effects of age and cognitive level (see Table 11). In the final model, the significant predictors were age, Ritualistic/Sameness, and Self-Injurious. Thus, younger age and higher scores on Ritualistic/Sameness and Self-Injurious subscales were associated with greater levels of Externalizing Behavior. However, cognitive level, Stereotypy, Compulsive, and Restricted Interests were not significant predictors of Externalizing Behavior.

**Table 11:** Three-Step Regression for Externalizing Behavior – Age, Cognitive Level, and RBS-R

Model	B	SE.B	β	*t*	*R* ^2^	∆*R*^2^	η_p_^2^
1 (Constant) Age	60.12 − 0.033	0.551 0.005	− 0.132	109.03* -6.93*	0.017	0.017	0.02
2 (Constant) Age Cognitive level	60.12 − 0.033 0.000	0.552 0.007 0.006	− 0.133 0.001	109.01* -4.92* 0.03	0.017	0.000	0.01 0.00
3 (Constant) Age Cognitive level R/S SIB STER COMP REST	53.46 − 0.048 0.016 6.51 6.96 0.99 − 0.55 0.15	0.601 0.006 0.005 0.458 0.560 0.446 0.451 0.282	− 0.195 0.074 0.332 0.235 0.049 − 0.026 0.012	90.60* -8.53* 2.92 14.22* 12.43* 2.22 -1.22 0.544	0.254	0.236	0.02 0.00 0.07 0.05 0.00 0.00 0.00
* F* (7, 2689) = 130.59*							

*Note.* R/S = Ritualistic/Sameness; SIB = Self-Injurious; STER = Stereotypy; COMP = Compulsive; REST = Restricted Interests

* p < .001

## Discussion

The purpose of this exploratory study was to identify patterns in RRBIs experienced by autistic children, based on sex, age, and cognitive level, and to explore the association between RRBIs and mental health problems. This study used individual RRBI subcategories rather than a total RRBI measure. Similar to the findings found in Siracusano et al. (2021) [18], results revealed no significant sex differences in the patterns of RBS-R subtypes between males and females. Thus, in this sample, males and females engaged in similar levels of each of the types of RRBIs. Although Frazier et al. (2014) [16] used the same sample and did report that females generally showed lower RRBIs assessed via the ADI-R and fewer restricted interests assessed via RBS-R, our findings may have diverged due to our use of a more stringent alpha level. Importantly, studies have shown that the types of RRBIs in females are more difficult to characterize as “atypical” (e.g., random behaviors) than those found in males (e.g., excessive lining up of toys) [39, 40]. The findings on RRBI sex differences should be interpreted with caution as the RRBI assessment measures have been normed using majority male samples, and thus may more accurately detect RRBIs in boys than in girls [41].

With respect to age differences, there were discrepancies found in the level of Ritualistic/Sameness and Stereotypy across age groups. Specifically, the middle childhood group engaged in significantly more Ritualistic/Sameness behaviors than the early childhood group, similar to patterns observed in Uljarević and colleagues (2022) [42]. This increase in Ritualistic/Sameness parallels the development of common fears and phobias in a typically-developing sample [43] and suggests that the increase in these behaviors reflects an underlying mechanism of self-regulation to reduce unpredictability in the environment [42]. Further, the early and middle childhood group both engaged in significantly more Stereotypy than the adolescent age group, similar to previous cross-sectional and longitudinal studies [23, 44]. This pattern is also found in non-autistic young children to promote the development of motor, language, and general cognitive functioning, which then shows a steep decline with age as more adaptive behaviors develop [4, 5]. There were no other significant age-related differences among Self-Injurious Behavior, Compulsive Behavior, or Restricted Interests. Overall, it can be concluded that there seem to be some clear age-related differences in the types and severities of RRBIs rated in this sample.

With respect to cognitive level differences, we found group differences in all subtypes of RRBIs. Those with higher cognitive level engaged in significantly more Ritualistic/Sameness than those with lower cognitive level. Previous research has identified Ritualistic/Sameness as a consistent deficit found in the ASD population, independent of individual characteristics such as sex, age, and cognitive level [44, 45]. However, Uljarević et al. (2022) [42] did find a small increase in insistence on sameness behaviors with increasing cognitive level scores. Similar to chronological age, higher Ritualistic/Sameness behaviors in higher functioning may represent capacity for self-regulation, a cognitively-driven construct. Self-regulation in autistic individuals serves to reduce fears of uncertainty and maintain consistency in day-to-day life by engaging in rituals [42]. In line with previous literature [21, 45, 46], Self-Injurious Behaviors, Stereotypy, Compulsive Behavior, and Restricted Interests were significantly more prevalent among those in the lower functioning groups. These findings provide supporting evidence that most RRBI subtypes tend to be less prevalent in individuals with higher cognitive level than those with lower cognitive level.

With the potential of RRBIs to interfere with daily functioning, learning, and well-being, identifying the patterns of specific RRBIs experienced at different developmental stages and when other mental health problems exist is important in clinical contexts [47]. Thus, we sought to examine the association between RRBIs and mental health problems, specifically internalizing and externalizing behaviors.

After controlling for the effects of age and cognitive level, our regression analyses revealed that young autistic individuals with a higher cognitive level who had more problems with Ritualistic/Sameness, Self-Injurious Behavior, and Stereotypy demonstrated high levels of internalizing behavior. Cognitive level was the strongest predictor of internalizing behavior in this regression. Further, higher cognitive level is generally associated with increased depression and anxiety in children and youth on the autism spectrum, as seen in a study by Mayes et al. (2011) [48]. This suggests that children with lower cognitive level may not experience such feelings or, more likely, parents reporting about their children with lower cognitive level may not recognize feelings of anxiety or depression in their children. Further, these findings add to the literature on the link between RRBIs and internalizing behaviors. Our findings are similar to previous studies that found that Ritualistic/Sameness [14] and Self-Injurious Behavior [14, 49] were positively associated with levels of anxiety and depression. To our knowledge, studies examining the association between Stereotypy and internalizing behaviors are sparse with only a number of studies demonstrating that toddlers with ASD and co-occurring anxiety demonstrated higher levels of Stereotypy than those with just ASD [50, 51]. These findings suggest that young children with higher cognitive levels and who show more problems with RRBIs exhibit more symptoms of internalizing behavior. Additionally, given the socially stigmatising nature of stereotypic behaviors [52], autistic individuals with high cognitive levels may feel distress when engaging in these behaviors or when attempting to inhibit these behaviors.

After controlling for the effects of age and cognitive level, we found that young autistic individuals who showed more problems with Ritualistic/Sameness and Self-Injurious Behavior demonstrated higher levels of externalizing behavior. Ritualistic/Sameness was the strongest predictor of externalizing behavior. Previous literature revealed that when individuals face interruptions or unexpected changes in routine that interfere with their execution of ritualistic/sameness behaviors, they may have meltdowns or display aggression [42]. Like internalizing behavior, young children seem to “grow” out of these externalizing behaviors. Guererra et al. (2019) [53] reported that younger children score higher on measures of aggressive behaviors, which make up our measure of externalizing behavior. These behaviors generally tend to decrease over time [54]. Similar to this study, more problems with Ritualistic/Sameness have been found to be associated with Oppositional Defiant Disorder severity in previous literature [14, 55]. Mazurek et al. (2013) [56] also found a significant association between self-injury and aggression, in line with the findings from this study. This could be explained as the emergence of reactive aggressive behavior when a child is interrupted from engaging in rituals or self-injury; however, further research is needed to elucidate these associations. Surprisingly, cognitive level did not significantly predict Externalizing Behavior. The Externalizing Behavior score, which only includes two subscale scores (i.e., Rule-Breaking Behavior and Aggressive Behavior) may not be comprehensive enough to detect cognitive level impacts. Research suggests that the measure of CBCL Externalizing Behavior may not be as sensitive to the types of problem behaviors experienced by autistic individuals who have lower cognitive levels [57]. Thus, further research is needed with measures validated for those with lower cognitive levels and with a more comprehensive assessment of externalizing behavior symptoms.

Overall, these findings suggest that, in addition to age and cognitive level, RRBIs are also strongly implicated in the presence of mental health problems in children and youth on the autism spectrum, accounting for about 20% of the variance. Specifically, Ritualistic/Sameness and Self-Injurious Behavior were predictive of both internalizing and externalizing behaviors; Stereotypy was only predictive of internalizing behavior. Thus, although both are lower-order RRBIs, Stereotypy and Self-Injurious Behavior were differentially related to mental health problems, suggesting different mechanisms of operation.

Although it is unclear whether RRBIs serve as coping strategies by individuals to help alleviate distress or whether RRBIs are a result of distress, the findings of this study have implications for clinicians. The use of RRBI subtypes instead of a total score or a broad categorization allows for a clearer understanding of distinct RRBI profiles, which may suggest different mechanisms of operation that can be used as targets for intervention. For example, based on the results of this study, it may be possible to reduce symptoms of anxiety by targeting Ritualistic/Sameness behaviors, or vice versa. This study highlights the associations between ritualistic/sameness, stereotypy, and self-injurious behaviors with co-occurring mental health problems. Thus, when assessing RRBIs in ASD, clinicians are encouraged to consider frequently co-occurring mental health issues and to design a holistic, individualized intervention that target both (e.g., behavioral intervention for ritualistic/sameness and psychotherapy for internalizing and externalizing behavior). It is important for RRBIs, specifically those that are perceived to be distressing to the autistic individual, to be treated early before becoming more fixed and challenging to manage, which may impact well-being and adaptive functioning [58, 59]. Lastly, RRBIs are heterogenous and may tap into varying underlying neural circuity [60]. Thus, identifying specific RRBI subtypes may allow researchers to identify more homogenous subgroups of autistic individuals, and to examine their underlying neurobiological underpinnings [9].

Although we had a large sample size with a wide range of cognitive abilities, families voluntarily participated in the study, and all had only one child on the autism spectrum (without major co-occurring mental health problems), making it difficult to ascertain generalizability. There were substantially more children than adolescents in the sample and more males than females. Thus, future studies are needed with equal representations of sex, particularly as the female ASD phenotype is becoming more recognized. This study used informant reports to assess RRBIs and mental health problems, which may not always be accurate as parents may have habituated to the atypicality of the behavior. Additionally, research suggests that the CBCL should be used with caution in children and youth on the autism spectrum as emotional and behavioral problems may be expressed differently than in TD children [60]. By using different methods of measuring RRBIs (e.g., observational) and psychopathology (e.g., self-report if possible; individual items of CBCL), future studies will be able to examine their association more accurately. Moreover, given the heterogenous nature of RRBIs, our cross-sectional design does not provide us with information on the presentation of RRBIs over time and associated changes in mental health. Thus, this study demonstrates the need for longitudinal studies to capture the lifetime trajectories of RRBIs and their association with mental health. The possibility of different developmental trajectories for subsets of children by cognitive level or sex might be a fruitful line of future research.

## Conclusions

It is important to disaggregate RRBIs into distinct behaviors given the heterogeneity within this construct and to obtain accurate data that may suggest mechanisms of RRBI operation [61]. This study revealed the differential associations of various RRBIs with individual characteristics and mental health problems. Given the differential associations with age and cognitive level, it is clear that RRBI types and severities are experienced differently across developmental levels. Additionally, this study revealed a positive association between ritualistic/sameness, self-injurious, and stereotypic behaviors with mental health problems, suggesting a substantial relationship between these RRBIs and mental health problems. Although directionality cannot be determined, these findings will allow for clinicians to design individualized treatment plans that incorporate mental health interventions with RRBI interventions to target potential mechanisms of operation (e.g., internalizing behavior maintaining stereotypy or vice versa) while considering an individual’s developmental level.

## Data Availability

The SSC dataset may be requested from the co-author, Dr. Adrienne Perry.

## List of abbreviations

ADI-R: Autism Diagnostic Interview–Revised
ADOS: Autism Diagnostic Observation Schedule
ASD: Autism Spectrum Disorders
ASEBA: Achenbach System of Empirically Based Assessment
CBCL: Child Behavior Checklist
H-RRBIs: Higher-order repetitive and restricted behaviors and interests
L-RRBIs: Lower-order repetitive and restricted behaviors and interests
RBS: Repetitive Behavior Scale
RBS-R: Repetitive Behavior Scale–Revised
RRBIs: Repetitive and Restricted Behaviors and Interests
SFARI: Simons Foundation Autism Research Initiative
SSC: Simons Simplex Collection
TD: Typically developing
